# Resistance to Both Chemotherapy and EGFR-TKI in Small Cell Lung Cancer With EGFR 19-Del Mutation: A Case Report

**DOI:** 10.3389/fonc.2020.01048

**Published:** 2020-07-17

**Authors:** Lingfei Wang, Fangyuan Dong, Jie Su, Guanjun Du, Yang Shao, Ying Liu, Xuequn He, Liubin Bao, Wei Wang, Xin Guo, Xi Wang

**Affiliations:** ^1^Department of Oncology, The 903rd Hospital of PLA, Hangzhou, China; ^2^Department of Breast Medicine, Liaoning Cancer Hospital, Cancer Hospital of China Medical University, Shengyang, China; ^3^Department of Pathology, The 903rd Hospital of PLA, Hangzhou, China; ^4^Nanjing Geneseeq Technology Inc., Nanjing, China; ^5^School of Public Health, Nanjing Medical University, Nanjing, China

**Keywords:** SCLC, EGFR 19-Del, EGFR-TKI, drug resistance, PTEN mutation

## Abstract

Epidermal growth factor receptor (EGFR) mutations are common in non-small cell lung cancers, but rare in small cell lung cancers (SCLCs). In previous reports, some SCLC patients with EGFR mutations could benefit from EGFR tyrosine kinase inhibitors (TKIs). In this study, we reported a case in which an SCLC patient with EGFR exon 19 deletion (19-Del) mutation did not benefit from EGFR-TKIs. Interestingly, the standard treatment strategies for SCLC also failed to control tumor progression. Moreover, we screened 43 SCLC patients in China and found that the frequency of EGFR mutations in Chinese SCLC patients was about 4.65% by next-generation sequencing (NGS). Collectively, this case illustrated a rare subtype of SCLCs which harbored EGFR mutations and was intrinsically resistant to standard treatments and EGFR-TKIs. We also tried to explore the mechanisms underlying drug resistance. The literature concerning SCLCs with EGFR mutations is reviewed.

## Introduction

Epidermal growth factor receptor (EGFR) mutations are found in 25–45.6% of Asian non-small cell lung cancer (NSCLC) patients and about 24% of white patients (1–3). It is a biomarker for the use of EGFR tyrosine kinase inhibitors (TKIs). However, EGFR mutations were rarely detected in small cell lung cancers (SCLCs) (4, 5). According to previous studies, 1.8% of 113 Italian SCLC patients and 4% of 122 Japanese SCLC patients had EGFR mutations (4, 6). In China, the patients with EGFR mutations accounted for 2.6–7.1% of SCLC patients (7–10).

SCLCs with EGFR mutations were reported to be sensitive to EGFR-TKIs (4, 11–13). EGFR-mutated SCLC patients tended to be female, non-smoker, and had limited disease (6, 14, 15). EGFR mutations may indicate a possible positive prognostic effect (6, 14, 15). Here, the patient we reported was also female and non-smoker, but her cancer was aggressive. The tumor metastasized rapidly to distant organs including brain, liver, adrenal gland, spinal cord, and vertebrae. Our case may suggest that EGFR mutations are not the only significant predictors for a positive outcome in SCLCs. More importantly, we also explored the possible mechanisms underlying tumor resistance to both chemotherapy and EGFR-TKIs in EGFR-mutated SCLCs.

## Genomic Analysis of Pure SCLC Tumors

**Figure 1:**
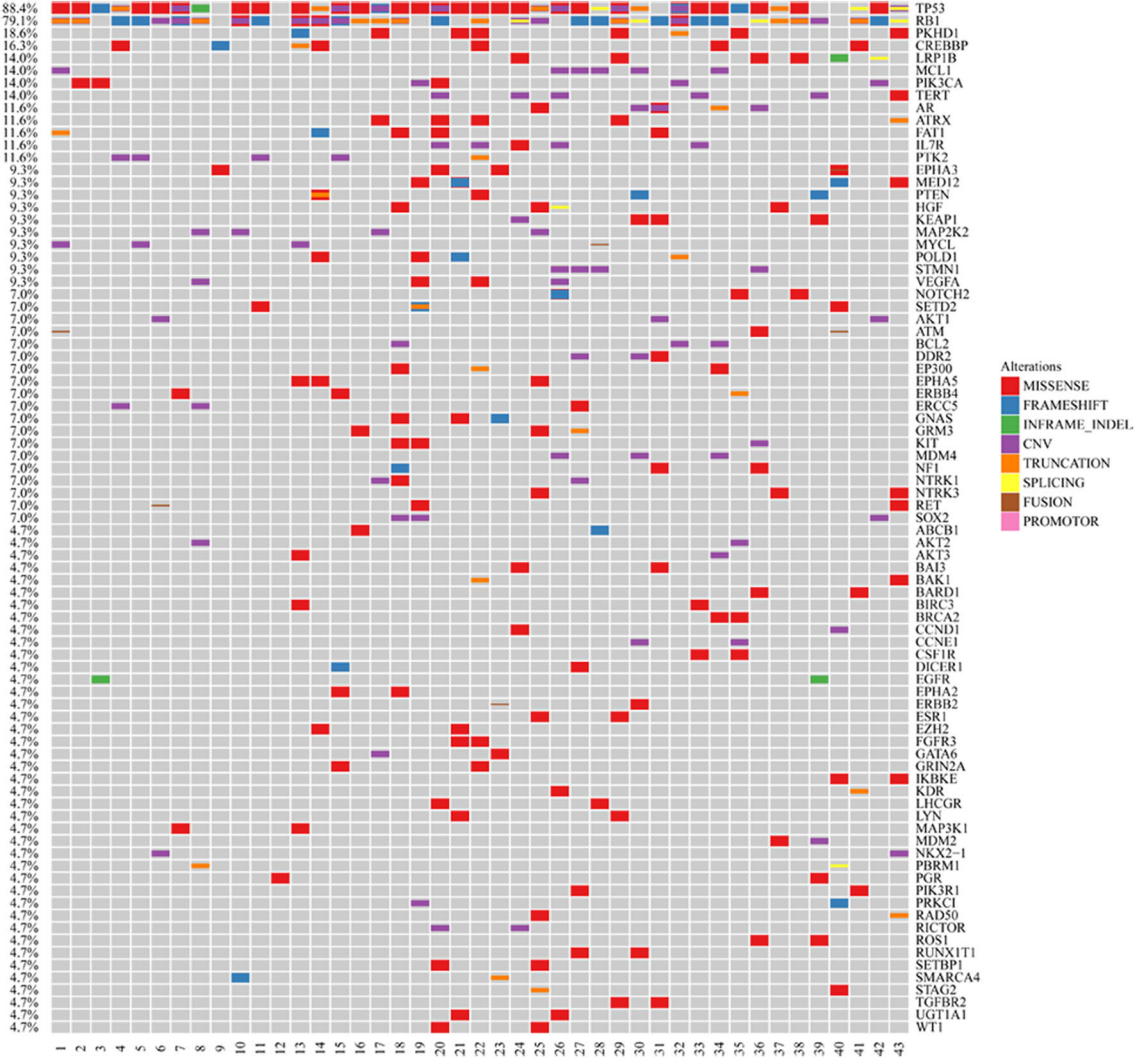
Genomic profiling of the 43 Chinese SCLC patients. The abscissa was tumor specimens, and the ordinate was gene names. Genetic alterations annotated according to the color panel on the right side of the image.

We excluded SCLC cases with adenocarcinoma components and collected 43 pure SCLC cases from hospitals in Zhejiang Province, China. The cases were identified as SCLC by pathological diagnosis according to the standard criteria of WHO classification. The median age of the study group was 62 years (range, 36–85). Seventy-seven percent of the cohort were male, and 63% of them had extended disease (Supplemental Table 1). The samples in these cases were subjected to next-generation sequencing (NGS) utilizing the Illumina HiSeq 4000 platform. The sequencing time ranged from July 2016 to September 2019. We found that the frequency of EGFR mutations in Chinese SCLC patients was 4.65% (Figure 1). Also, we demonstrated the genomic profiling of the 43 Chinese SCLC patients (Figure 1).

## Case Presentation

**Figure 2:**
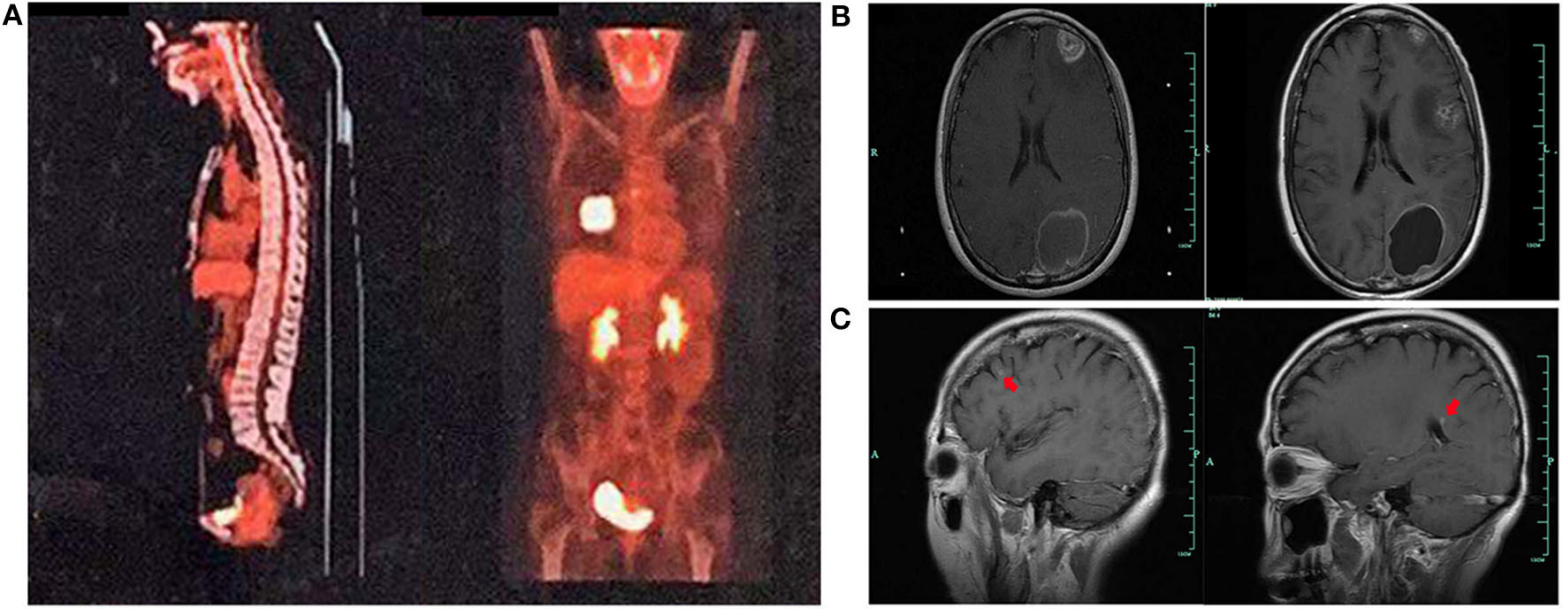
Tumor lesions detected at various times. PET-CT showing a mass in the middle lobe of the right lung **(A)**. Tumor lesions in the brain before and after EP therapy **(B)**. New masses in the brain after EP therapy **(C)**.

**Figure 3:**
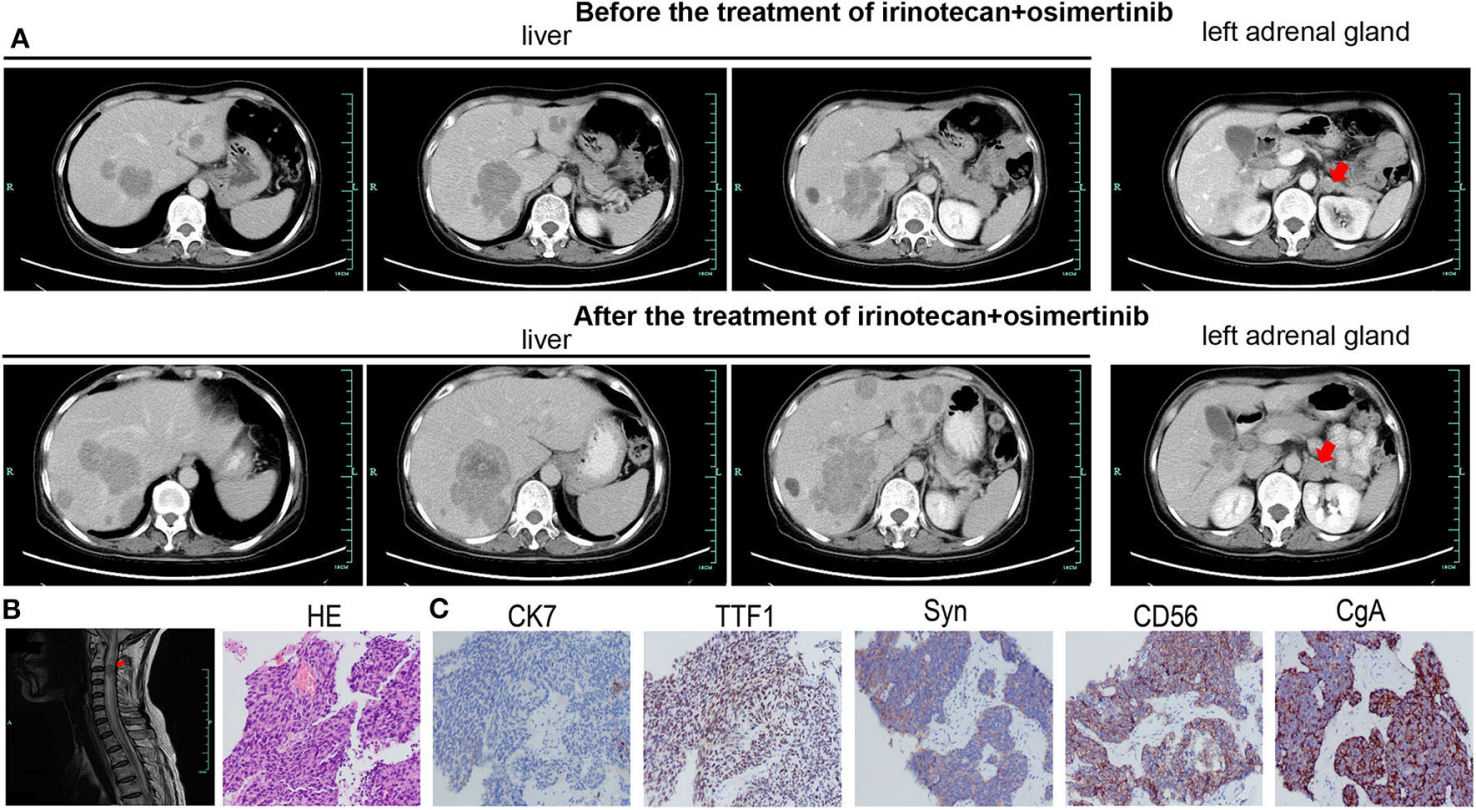
The imageological and cytological examinations of tumor metastases. Tumor metastases in the liver **(A)**, left adrenal **(A)**, and spinal cord **(B)** before irinotecan plus osimertinib, and enlarged metastatic lesions in the liver **(A)** and left adrenal **(A)** after treatment. **(C)** The HE and IHC staining of biopsy specimens in the case. Typical for SCLC, IHC was strongly positive for TTF1, Syn, CD56, and CgA.

A 59-year-old woman without smoking history presented with intermittent cough and dizziness. Computed tomography (CT) of the chest and brain revealed a right middle lobe lung mass and a left occipital lobe brain metastasis. Magnetic resonance imaging (MRI) of the brain demonstrated an enhancing mass in the left occipital lobe (1.7 × 1.4 cm). Bronchoscopic examination revealed a lesion occluding the right lateral middle lobe bronchus. Transbronchial biopsy of the right middle lobe mass was positive for small cell carcinoma. The histological H&E examination of biopsy specimens showed small and poorly differentiated cells with round or oval nuclei. The biopsies were devoid of any evidence of non-small-cell components. Histopathology reported a TTF-1-positive, Syn-positive, Ki-67 (60–70%), small cell carcinoma of the lung. Whole-body positron emission tomography computed tomography (PET-CT) identified areas of abnormal metabolism in the right lung, right hilum, and subcarinal lymph nodes (Figure 2A), as well as the left occipital lobe of the brain. The bone scan was negative for evidence of metastatic lesions. Two months after diagnosis, she was referred to our department. Brain MRI showed the metastatic tumor in the left occipital lobe dramatically increasing to 4.8 × 3.8 cm (Figure 2B). Meanwhile, four new masses were found in brain. As standard therapy for extensive-stage small cell carcinoma, the patient received etoposide and cisplatin (EP) chemotherapy with a poor clinical response. After four cycles of EP treatment, new metastatic lesions were detected in the right brain (Figure 2C). To control tumor progression, she received CyberKnife radiosurgery for the treatment of lung neoplasm, followed by a whole-brain radiotherapy (WBRT) for brain metastases. After WBRT, the patient displayed symptomatic disease progression with multiple new liver, left adrenal gland, and spinal cord metastases (Figure 3A,B). An ultrasound-guided percutaneous liver biopsy was performed. Re-biopsy of the metastases in the liver confirmed small cell histology and revealed SCLC evident by positive expressions of CD56, TTF-1, CgA, and synaptophysin (Figure 3C). The tissue re-biopsy samples and blood samples were subjected to next-generation sequencing. As shown in Table 1, genotype disclosed an EGFR exon 19 deletion (19-Del) mutation and PTEN frameshift mutation (p.K237Cfs^*^17). She subsequently received two cycles of irinotecan in combination with 1 month of third-generation EGFR-TKI osimertinib. However, the tumor dramatically progressed with enlargement of liver metastases (Figure 3A). After that, her disease also failed to respond to anlotinib plus pembrolizumab and anlotinib plus Tiggio. Finally, with fast tumor progression, she died 1 month later.

**Table 1:** Genetic alterations in the case identified by NGS.

**Genes**	**Alternations**	**Nucleotide change**
ARID1B	p.S36F Missense mutation in exon 1	c.107C>T (p.S36F)
ASXL1	p.E332D Missense mutation in exon 11	c.996G>C (p.E332D)
EGFR	p.E746_A750del Non-shift code deletion mutation in exon 19	c.2235_2249delGGAATTAAGA GAAGC (p.E746_A750del)
KEAP1	p.A392T Missense mutation in exon 3	c.1174G>A (p.A392T)
MDM2	Amplification	-
PDCD1	p.R231 Truncation in exon 5	c.691C>T (p.R231*)
PGR	p.Q78H Missense mutation in exon 1	c.234G>T (p.Q78H)
PTEN	p.K237Cfs*17 7 Frameshift mutation in exon 7	c.709_715delAAGTTCA (p.K237Cfs*17)
RB1	Single copy number missing	-
ROS	p.G1809E Missense mutation in exon 33	c.5426G>A (p.G1809E)
SMAD4	c.1447+2dupT Shear mutation in Intron 11	c.1447+2dupT
TERT	Amplification	-

–*, not applicable*.

## Discussion

SCLCs are often seen as aggressive cancers which are sensitive to chemotherapy and shrink at the initial stage and then relapse soon with chemoresistance. Currently, the exact mechanism underlying chemoresistance is still unknown. As previous studies have shown, the mutations in CSMD3/PCLO/RYR1/EPB41L3 may predict resistance to etoposide (16). However, none were detected in our case. In our case, PTEN mutation was detected. PTEN is a tumor suppressor in the PI3K/AKT pathway. The continuous activation of the PI3K/AKT pathway is a pivotal chemoresistance factor in SCLCs. PTEN could sensitize the tumor to chemotherapy including etoposide and cisplatin by inhibiting the PI3K/AKT signaling pathway (17, 18). The efforts to combine adenoviral PTEN gene therapy with cisplatin chemotherapy could enhance tumor suppression in SCLC (19). Thus, PTEN mutation may account for chemoresistance in our case by aberrant activation of the PI3K/AKT pathway.

In most previous studies, EGFR mutations in lung cancer, even in SCLC patients, are responsive to EGFR-TKIs (4, 11, 13, 20). It is still controversial whether EGFR-mutated SCLCs have adenocarcinoma components. Some researchers propose that EGFR mutations do exist but are just rare in SCLCs (4, 15). In our case, the patient received biopsy twice and the pathological examinations were almost identical. We did not find any pathological evidence supporting adenocarcinoma. Different from the favorable response to EGFR-TKIs in adenocarcinoma, the tumor in our case proved to be resistant to EGFR-TKI. The mechanisms of resistance to EGFR-TKIs are widely discussed. Le et al. discussed that the lack of response to EGFR-TKIs in EGFR-mutated *de novo* SCLC might be attributable to the lack of EGFR expression (21). Mutations of KRAS or BRAF might also be correlated with resistance to EGFR-TKIs in EGFR-mutated *de novo* SCLC patients (21, 22). Many researchers pointed out that EGFR and its downstream pathway were involved with primary resistance to EGFR-TKIs (23). Mutations in the EGFR downstream genes including PIK3CA and PTEN might confer resistance to EGFR-TKIs (22–27). A study using a genetic mouse model showed that PTEN inactivation advanced SCLC, suggesting that treatment targeting PTEN may be effective for a subset of SCLC patients (28). PTEN loss-of-function mutation resulted in poor PFS and OS in patients with EGFR mutations, and it was an independent predictor for short PFS in patients with EGFR-TKI treatment (23–25). Furthermore, in the 43 sequenced SCLC cases, one EGFR-mutated SCLC without PTEN mutation was responsive to EGFR-TKI gefitinib. Thus, our case suggests that PTEN may also play a key role in the poor outcome with EGFR-TKI treatment in EGFR-mutated SCLC patients.

Few studies have focused on the potential mechanisms of tumor resistance to both EGFR-TKIs and chemotherapy in EGFR-mutated *de novo* SCLC patients. Our case is the first one to propose that in this rare subtype of SCLCs, PTEN dysfunction may play a vital role in the instinct resistance to both chemotherapy and EGFR-TKIs. Another case resistant to both EGFR-TKIs and chemotherapy was reported by Varghese et al. (28). EGFR 19-Del and PIK3CA mutations coexisted in their case (28). Therefore, the aberrant activation of the PI3K/AKT pathway may be involved in the resistance of EGFR-mutated SCLC to both chemotherapy and EGFR-TKIs. Large sample size and further studies in EGFR-mutated SCLCs are needed to validate our findings.

## Conclusion

We have reported a *de novo* SCLC case with EGFR 19-Del which was innately resistant to both chemotherapy and EGFR-TKI. The resistance to EGFR-TKIs and chemotherapy may be attributable to PTEN mutation. Moreover, we presented the frequencies of EGFR mutations and other genetic mutations in Chinese SCLC patients.

## Ethics Statement

The studies involving human participants were reviewed and approved by Ethical Committee of the 903rd Hospital of PLA. The patients/participants provided their written informed consent to participate in this study. Written informed consent was obtained from the individual(s) for the publication of any potentially identifiable images or data included in this article.

## Author Contributions

LW wrote this article. LW and FD were involved in diagnostic flow and patient follow-up. JS was a licensed pathologist and proposed the images of pathological examination. GD and YS contributed to the data collection and analysis of 43 pure SCLC cases using NGS technology. The manuscript was edited by XW. LW, FD, JS, GD, YS, YL, XH, LB, WW, and XG were involved in the interpretation of published data. All authors read and gave their final approval of the version to be published.

## Conflict of Interest

GD and YS were employed by the company Nanjing Geneseeq Technology Inc., Nanjing, China. The remaining authors declare that the research was conducted in the absence of any commercial or financial relationships that could be construed as a potential conflict of interest.

## Supplementary Material

Supplemental Table 1 The clinical characteristics of the 43 Chinese SCLC patients.

## References

[1] Graham RP Treece AL Lindeman NI Vasalos P Shan M Jennings LJ. Worldwide frequency of commonly detected EGFR mutations. Arch Pathol Lab Med. (2018) 142:163–7. 10.5858/arpa.2016-0579-CP 29106293

[2] Lindeman NI Cagle PT Beasley MB Chitale DA Dacic S Giaccone G. Molecular testing guideline for selection of lung cancer patients for EGFR and ALK tyrosine kinase inhibitors: guideline from the College of American Pathologists, International Association for the Study of Lung Cancer, and Association for Molecular Pathology. J Mol Diagn. (2013) 15:415–53. 10.1097/JTO.0b013e318290868f 23562183

[3] Girard N Sima CS Jackman DM Sequist LV Chen H Yang JC. Nomogram to predict the presence of EGFR activating mutation in lung adenocarcinoma. Eur Respir J. (2012) 39:366–72. 10.1183/09031936.00010111 21778168

[4] Tatematsu A Shimizu J Murakami Y Horio Y Nakamura S Hida T. Epidermal growth factor receptor mutations in small cell lung cancer. Clin Cancer Res. (2008) 14:6092–6. 10.1158/1078-0432.CCR-08-0332 18829487

[5] Shigematsu H Gazdar AF. Somatic mutations of epidermal growth factor receptor signaling pathway in lung cancers. Int J Cancer. (2006) 118:257–62. 10.1002/ijc.21496 16231326

[6] Bordi P Tiseo M Barbieri F Bavieri M Sartori G Marchetti A. Gene mutations in small-cell lung cancer (SCLC): results of a panel of 6 genes in a cohort of italian patients. Lung Cancer. (2014) 86:324–8. 10.1016/j.lungcan.2014.10.002 25453846

[7] Wang Z Jiang Z Lu H Molecular genetic profiling of small cell lung carcinoma in a chinese cohort. Transl Cancer Res. (2019) 8:255–61. 10.21037/tcr.2019.01.26 PMC8798498 35116754

[8] Shiao TH Chang YL Yu CJ Chang YC Hsu YC Chang SH. Epidermal growth factor receptor mutations in small cell lung cancer: a brief report. J Thorac Oncol. (2011) 6:195–8. 10.1097/JTO.0b013e3181f94abb 21178714

[9] Hu J Wang Y Zhang Y Yu Y Chen H Liu K. Comprehensive genomic profiling of small cell lung cancer in chinese patients and the implications for therapeutic potential. Cancer Med. (2019) 8:4338–47. 10.1002/cam4.2199 31199602 PMC6675718

[10] Tang H Zhang J Hu X Xu Y Dong B Wang J EGFR mutations in small cell lung cancer (SCLC): genetic heterogeneity and prognostic impact. J Thorac Oncol. (2017) 1:S710–S71. 10.1016/j.jtho.2016.11.936

[11] Okamoto I Araki J Suto R Shimada M Nakagawa K Fukuoka M. EGFR mutation in gefitinib responsive small-cell lung cancer. Ann Oncol. (2006) 17:1028–9. 10.1093/annonc/mdj114 16357019

[12] Zakowski MF Ladanyi M Kris MG. Memorial sloan-kettering cancer center lung cancer oncogenome group. EGFR mutations in small-cell lung cancers in patients who have never smoked. N Engl J Med. (2006) 355:213–5. 10.1056/NEJMc053610 16837691

[13] Araki J Okamoto I Suto R Ichikawa Y Sasaki J. Efficacy of the tyrosine kinase inhibitor gefitinib in a patient with metastatic small cell lung cancer. Lung Cancer. (2005) 48:141–4. 10.1016/j.lungcan.2004.10.012 15777982

[14] Cardona AF Rojas L Zatarain-Barrón ZL Ruiz-Patiño A Ricaurte L Corrales L. Multigene mutation profiling and clinical characteristics of small-cell lung cancer in never-smokers vs. heavy smokers (Geno1.3-CLICaP). Front Oncol. (2019) 9:254. 10.3389/fonc.2019.00254 31058075 PMC6481272

[15] Siegele BJ Shilo K Chao BH Carbone DP Zhao W Ioffe O. Epidermal growth factor receptor (EGFR) mutations in small cell lung cancers: two cases and a review of the literature. Lung Cancer. (2016) 95:65–72. 10.1016/j.lungcan.2016.02.012 27040854 PMC5524560

[16] Qiu Z Lin A Li K Lin W Wang Q Wei T. A novel mutation panel for predicting etoposide resistance in small-cell lung cancer. Drug Des Devel Ther. (2019) 13:2021–41. 10.2147/DDDT.S205633 31417239 PMC6594009

[17] Mayo LD Dixon JE Durden DL Tonks NK Donner DB. PTEN protects p53 from Mdm2 and sensitizes cancer cells to chemotherapy. J Biol Chem. (2002) 277:5484–9. 10.1074/jbc.M108302200 11729185

[18] Wu H Cao Y Weng D Xing H Song X Zhou J. Effect of tumor suppressor gene PTEN on the resistance to cisplatin in human ovarian cancer cell lines and related mechanisms. Cancer Lett. (2008) 271:260–71. 10.1016/j.canlet.2008.06.012 18657898

[19] Li D Zhang Y Xie Y Xiang J Zhu Y Yang J. Enhanced tumor suppression by adenoviral PTEN gene therapy combined with cisplatin chemotherapy in small-cell lung cancer. Cancer Gene Therapy. (2013) 20:251–9. 10.1038/cgt.2013.14 23470565

[20] Asai N Ohkuni Y Matsuda M Kaneko N. Small-cell lung cancer with epidermal growth factor receptor mutation: case report and review of literature. Indian J Cancer. (2014) 51:384–5. 10.4103/0019-509X.146753 25494153

[21] Le X Desai NV Majid A Karp RS Huberman MS Rangachari D. *De novo* pulmonary small cell carcinomas and large cell neuroendocrine carcinomas harboring EGFR mutations: lack of response to EGFR inhibitors. Lung Cancer. (2015) 88:70–3. 10.1016/j.lungcan.2015.02.003 25700797 PMC4355318

[22] Zhong J Li L Wang Z Bai H Gai F Duan J. Potential resistance mechanisms revealed by targeted sequencing from lung adenocarcinoma patients with primary resistance to epidermal growth factor receptor (EGFR) tyrosine kinase inhibitors (TKIs). J Thorac Oncol. (2017) 12:1766–78. 10.1016/j.jtho.2017.07.032 28818608

[23] Kim HR Cho BC Shim HS Lim SM Kim SK Chang J. Prediction for response duration to epidermal growth factor receptor-tyrosine kinase inhibitors in EGFR mutated never smoker lung adenocarcinoma. Lung cancer. (2014) 83:374–82. 10.1016/j.lungcan.2013.12.011 24468202

[24] Wang F Diao XY Zhang X Shao Q Feng YF An XJ. Identification of genetic alterations associated with primary resistance to EGFR-TKIs in advanced non-small-cell lung cancer patients with EGFR sensitive mutations. Cancer Commun (Lond). (2019) 39:7. 10.1186/s40880-019-0354-z 30823937 PMC6397445

[25] Li T Yang Y Li X Xu C Meng L. EGFR- and AKT-mediated reduction in PTEN expression contributes to tyrphostin resistance and is reversed by mTOR inhibition in endometrial cancer cells. Mol Cell Biochem. (2012) 361:19–29. 10.1007/s11010-011-1082-0 21952748

[26] Varghese AM Zakowski MF Yu HA Won HH Riely GJ Krug LM. Brief report: small cell lung cancers in patients who never smoked cigarettes. J Thorac Oncol. (2014) 9:892–6. 10.1097/JTO.0000000000000142 24828667 PMC4199745

[27] Petricevic B Tay RY Califano R Treatment resistant *de novo* epidermal growth factor receptor (EGFR)-mutated small cell lung cancer. Eur Oncol Hematol Rev. (2018) 14:84–6. 10.17925/EOH.2018.14.2.84

[28] Cui M Augert A Rongione M Conkrite K Parazzoli S Nikitin AY. PTEN is a potent suppressor of small cell lung cancer. Mol Cancer Res. (2014) 12:654–9. 10.1158/1541-7786.MCR-13-0554 24482365 PMC4020961

